# Chest-CT-based radiomics feature of epicardial adipose tissue for screening coronary atherosclerosis

**DOI:** 10.1007/s00380-024-02479-2

**Published:** 2024-11-14

**Authors:** Qin Wei, Yanling Chen, Deqing Yuan, Fumei Nie, Jian Li, KeBing Yu, Chengwei Zhang

**Affiliations:** 1 Health Management Centre, The Second Affiliated Hospital of Chengdu Medical College, Nuclear Industry 416 Hospital, Chengdu, 610051 China; 2 Occupational Diseases Section, The Second Affiliated Hospital of Chengdu Medical College, Nuclear Industry 416 Hospital, Chengdu, 610051 China; 3 Radiology department,, The Second Affiliated Hospital of Chengdu Medical College, Nuclear Industry 416 Hospital, Chengdu, 610051 China; 4 Cardiovascular Medicine, The Second Affiliated Hospital of Chengdu Medical College, Nuclear Industry 416 Hospital, Chengdu, 610051 China

**Keywords:** Epicardial adipose tissue, Radiomics feature, Coronary atherosclerosis stenosis, Subject operating characteristic curves

## Abstract

**Background and aims:**

This study aims to investigate the diagnostic value of chest-CT epicardial adipose tissue (EAT) radiomics feature in coronary atherosclerotic stenosis.

**Methods:**

Clinical data from 215 individuals who underwent coronary angiography and chest-CT scan from January to July 2022 at our institution were retrospectively analyzed. Based on the coronary angiography results, the total population, men, and women were divided into the CAD group and non-CAD group. radiomics feature of EAT at the level of the bifurcation of the left-main coronary artery on the transverse level of chest CT were measured. The features contain both first-order feature and shape-order feature.The differences between groups were analyzed using the *t* test or Chi-square test. The diagnostic efficacy of each parameter in diagnosing atherosclerotic stenosis of coronary arteries was assessed by plotting the receiver operating characteristic (ROC) curve.

**Results:**

First-order features: Mean, IntDen, Median, and RawIntDen; shape-order features: Area, Perim, Round, and BSA index; and clinical index: HbA1c showed statistical significance between the CAD group and the non-CAD group. The ROC curve analysis demonstrated high diagnostic efficacy, with the best for diagnostic efficacy being Median for the first-order feature parameter (AUC, 0.753; *95%* confidence interval [*CI*], 0.689–0.817; *t* = 4.785, *p* < 0.001), Round for the shape-order feature (AUC, 0.775; *95% CI*, 0.714–0.836;* t* = 7.842, *p* < 0.001), and HbA1c for the clinical index (AUC, 0.797; *95% CI*, 0.783–0.856; *t* = 6.406, *p* < 0.001). After dividing the participants into male and female subgroups, the best diagnostic efficacy was observed with the BSA index for men (AUC, 0.743; *95% CI*, 0.656–0.829; *t* = 5.128, *p* < 0.001) and Round for women (AUC, 0.871; *95% CI*, 0.793–0.949; *t* = 7.247, *p* < 0.001).

**Conclusions:**

Median, Round in radiomics feature of EAT on chest CT may play a role in the assessment of coronary atherosclerotic stenosis.

## Introduction

Epicardial adipose tissue (EAT) shares the same embryonic origin as intra-abdominal fat. It wraps around the myocardium and coronary arteries, sharing the blood supply and providing positive effects such as energy storage, protection, heating, and immune support [1, 2]. However, EAT can also be detrimental to the body, as it can directly cause damage through paracrine or vascular secretion of pro-inflammatory and pro-fibrotic cytokines, leading to coronary atherosclerosis [3, 4], atrial fibrillation, and heart failure [5, 6].In recent years, researchers have focused on characterizing the size, volume, and vascularity of EAT. The relationship between the volume, density, and attenuation index of EAT and coronary atherosclerosis has become a popular research topic [7–9]. Nevertheless, studies on radiomics feature for EAT quantification have been scarce [10].EAT is smaller and more numerous than abdominal adipocytes, and its stromal fibrosis and vascularization result in diverse cellular components. radiomics feature offers a quantitative and objective assessment of the heterogeneity of EAT cells, which proves useful in identifying the cellular characteristics of EAT. This study aims to utilize radiomics feature to observe the cellular and morphological characteristics of EAT in patients with varying degrees of coronary artery stenosis, in order to assess the predictive value of this non-invasive imaging method for coronary artery stenosis. In this study, the cases were divided into CAD group and non-CAD group according to the coronary angiography (CAG) results, and radiomics feature was performed on the EAT of chest-CT plain transverse images of the two groups to obtain grey scale histograms and some area-related to assess its predictive efficacy on coronary artery stenosis [11] and to provide reference for clinical diagnosis.

## Methods

### Object of the study

This retrospective analysis included 215 participants from 1st January to 31st July 2022, who had a mean age of (66.09 ± 11.72) years, including 123 males and 92 females. These data were collected for the purpose of this study and the participants underwent CAG and chest-CT plain film scanning at China National Nuclear Corporation 416 Hospital: Chengdu Medical College Second Affiliated Hospital. The total population was divided into two groups based on the results of CAG: the CAD group, comprising 97 cases with ≥ 50% stenosis of the coronary artery, and the non-CAD group, comprising 118 cases with coronary artery stenosis < 50% or no coronary artery stenosis. Among these participants, 63 males were in the CAD group, while 60 were in the non-CAD group, and 34 females were in the CAD group, with 58 in the non-CAD group.

Exclusion criteria: (1) participants without complete medical history, biochemical test results, and chest-CT data. (2) Participants with a history of cardiac surgery, including coronary artery bypass grafting and cardiac valvuloplasty. (3) Participants with a history of severe cardiac valvular disease, cardiomyopathy, and pericardial effusion. (4) Participants with acute infections. (5) Participants with autoimmune diseases.

Statement of assurance: (1) The study protocol conforms to the ethical guidelines of the 1975 Declaration of Helsinki. (3) The study protocol has been priorly approved by the Institution’s ethics committee on research on humans.

### Instruments and methods

An ICT Brilliance 128-row 256-slice CT scanner (Philips, The Netherlands) was used for this study, employing a tube voltage of 120 kVp, a tube current of 220 mA, a pitch of 1.375, a scanning thickness of 2.5 mm, and an image matrix of 512 × 512. Both standard algorithmic and non-algorithmic reorganization of the images were performed, with a reorganization layer thickness and spacing between layers set at 1.25 mm each. The images were analyzed using a soft tissue window with a window width of 350 HU and a window position of 35 HU. The scanning process covered the region from the thoracic inlet to the bottom of the lungs, with the patient completing the scan while holding their breath after one deep inspiratory breath. Chest-CT scans with thin-layer reconstruction were performed in this study without ECG gating, and one week after the chest-CT, CAG was conducted.

CAG was conducted via the Seldinger puncture method with transradial artery access. Based on the American Heart Association Coronary Angiography Stenosis Evaluation Criteria [12], patients with ≥ 50% coronary stenosis were classified into the coronary CAD group, while those with < 50% coronary stenosis or no coronary stenosis formed the non-CAD group. Furthermore, the left ventricle was divided into subgroups based on gender, consisting of the male CAD group and non-CAD group, and the female CAD group and non-CAD group.

### Clinical data collection

Age was calculated from the identity card number, while height, weight, and blood pressure measurements were taken by the cardiology nurse. The nurse also conducted tests for C-reactive protein (CRP), total cholesterol (TC), triglyceride (TG), fasting plasma glucose (FPG), glycosylated hemoglobin Alc (HbA1c), uric acid (UA), lipoprotein A (LpA), and apolipoprotein a (ApoA). In addition, the laboratory examined other biochemical indexes, including TC, TG, FPG, HbA1c, UA, LpA, and ApoA. Left ventricular ejection fraction (LVEF) was determined through echocardiography, while the degree of coronary artery stenosis was based on cardiologists’ post-CAI records and clinical diagnosis.

### Image segmentation and radiomics feature parameter acquisition

**Fig. 1:**
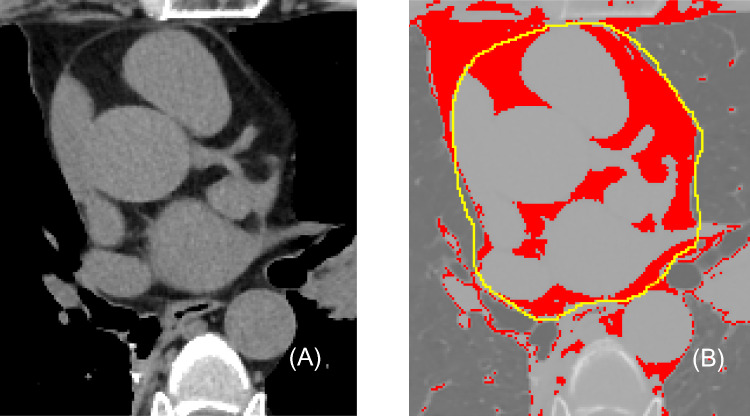
EAT left coronary bifurcation level outlining ROI. **A** Sixty-four -year-old male, LCX stenosis 60%; a EAT left-main coronary bifurcation level. **B**:Yellow line represents manually outlined EAT, red within yellow line is EAT area

**Fig. 2:**
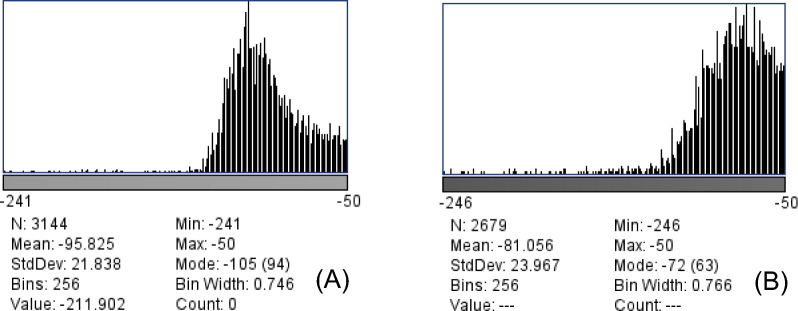
Gray-scale histogram of the EAT ROI. **A** Sixty-four -year-old male with 60% LCX stenosis.Gray-scale histogram waveform peaks to the left. **B** Fifty-nine-year-old male coronary artery without stenosis, gray-scale histogram waveform peaks to the right (Threshold range of less than Min-250 is due to software value interval)

The enrolled patients underwent Philips 128-row ICT chest scanning, and axial images were selected at the level of the bifurcation of the main trunk of the thin left coronary artery and saved in DICOM (Digital Imaging and Communications in Medicine) format. ImageJ (Image J, version 1.3.4.11. National Institutes of Health, Bethesda, Maryland) software was used to set the image threshold from – 250 HU to – 50 HU, while carefully avoiding pericardial fat along the visible cardiac fibrous membrane [13]. The region of interest (ROI) was obtained by manually outlining the edge of the EAT [14] (Fig. 1). First-order features were outlined, measured by two associate radiologists, each with over 20 years of experience in radiology (Fig. 2) were classified into two groups based on their significance (Table 1): first-order features i.e., gray-scale histogram parameters(Mean, StdDev, Mode, IntDen, Median, Skew, Kurt, RawIntDen, AR, Solidity), and shape (Area, BSA index, Perim, Circ, Area%, Round). The were then averaged, and grayscale values, except Skew, were taken as absolute values. Due to the nature of the measurements being parameters of fat, many feature values are negative. However, the negative sign in adipose tissue has a different meaning compared to the algebraic negative sign. This discrepancy can influence calculations, potentially resulting in contrary statistical outcomes. Therefore, with the exception of skewness, all negative values have been converted to their absolute values. BSA index were calculated by dividing the EAT area by the BSA value, which was obtained using the “Chinese general formula” based on height and weight: BSA = 0.0061 × height(cm) + 0.0124 × weight(kg) – 0.0099 [15].

**Table 1:** The meaning of each feature

Feature	Meaning	Feature	Meaning
Mean:	The average intensity value of pixels in the image	Skew**:**	Measure of the asymmetry of the intensity distribution
StdDev	Measure of the spread or variation of pixel intensities	Kurt	Measure of the peakedness or flatness of the intensity distribution
Mode	The most frequently occurring intensity value	RawIntDen	Similar to IntDen but without subtracting background intensity
IntDen	Total sum of all pixel intensities in the image	AR	Ratio of major axis length to minor axis length of the region
Median**:**	The middle intensity value when all pixel values are sorted	Solidity	Measure of the ratio of pixels in the convex hull to the pixels in the region

### Consistency evaluation

CT images of 40 patients were randomly selected for intra- and inter-group consistency assessment by two associate radiologists. In the intra-group consistency assessment, the first radiologist outlined the ROI and obtained the radiomics feature following specific steps. Afterward, the same method and steps were repeated within a 2-week interval, and the radiomics features of the 40 patients were extracted from both sets of data for intra-group correlation analysis. For the inter-group correlation assessment, the second radiologist outlined the CT images of the same 40 patients using the same methods and steps as the first radiologist. The radiomics feature obtained by the second radiologist were then compared with the ones extracted by the first radiologist for the initial analysis. The results showed a mean intra-group correlation coefficient (ICC) value of 0.93 (ranging from 0.785 to 1.000, *p* < 0.001) and a mean inter-group correlation coefficient of 0.91 (ranging from 0.732 to 1.000, *p* < 0.001).

### Statistical analysis

SPSS 19.0 software was utilized for data analysis. For metric data that followed a normal or near-normal distribution, the mean ± standard deviation was used for expression, and a *t* test was employed to compare between groups in the study design. Meanwhile, count data was presented using frequency and percentage, and the comparison between groups was conducted using the Chi-square test. Furthermore, clinical indicators and textural parameter indexes that exhibited statistically significant differences in the inter-group comparisons were depicted on the subjects’ operating characteristic ROC. For the clinical indicators and radiomics parameter indicators with statistically significant differences between the groups, ROC curves were plotted, and the area under the ROC curve (AUC) was calculated. Statistical significance was set at *p* < 0.05.

## Results

### Comparison of general conditions

The CAD group consisted of 97 participants (63 males and 34 females) with a mean age of 67.69 ± 12.99 years, while the non-CAD group included 118 participants (60 males and 58 females) with a mean age of 64.77 ± 10.49 years. Although there was no statistically significant difference in age between the two groups (*p* > 0.05), a significant difference was observed in gender composition (*p* < 0.05).

### Comparison of clinical data between the CAD group and the non-CAD group

Statistically significant differences (*p* < 0.05) were observed in gender, FPG, HbA1c, and LpA between the CAD group and the non-CAD group. Further analysis revealed that the male CAD group had significant differences (*p* < 0.05) with the non-CAD group in CRP and HbA1c levels. In addition, the female CAD group showed statistically significant differences (*p* < 0.001) compared to the non-CAD group in terms of HbA1c levels. Please refer to Table 2 for details.

**Table 2:** Comparison of clinical indicators between the stenosis group and the control group

	CRP (mg/L)	TC (mmol/L)	TG (mmol/L)	FPG (mmol/L)	HbA1c (mmol/mol)	UA (umol/L)	LpA (mmol/L)	ApoA (g/L)	SBP (mmHg)	DBP (mmHg)	LVEF (%)
Total population											
Stenosis group (97 cases)	4.55 ± 8.46	4.26 ± 1.31	1.79 ± 1.15	7.60 ± 3.74	6.93 ± 1.69	357.05 ± 95.48	212.61 ± 255.61	1.39 ± 0.28	134.08 ± 22.13	79.16 ± 14.45	64.71 ± 10.507
Control group (118 cases)	3.29 ± 5.79	7.29 ± 30.62	2.03 ± 1.38	6.58 ± 2.09	5.67 ± 1.02	338.09 ± 86.47	138.94 ± 198.51	2.28 ± 8.43	135.57 ± 18.44	79.95 ± 14.15	64.41 ± 9.11
*t*-value or Z-value	1.294	0.974	1.384	2.381	6.406	1.526	2.313	1.039	0.537	0.401	0.225
*p* value	0.197	0.413	0.168	0.019	< 0.001	0.128	0.022	0.3	0.592	0.689	0.822
Male											
Stenosis (63 cases)	5.753 ± 10.215	4.166 ± 1.1	1.922 ± 1.26	7.301 ± 3.371	6.819 ± 1.576	372.27 ± 92.068	200.484 ± 257.346	1.3248 ± 0.237	134.81 ± 21.22	79.89 ± 14.60	63.79 ± 10.81
Control (60 cases)	2.923 ± 4.353	9.994 ± 42.93927	2.255 ± 1.57	6.788 ± 2.469	5.7015 ± 0.745	368.057 ± 75.808	124.217 ± 166.03	1.406 ± 0.277	134.93 ± 18.99	80.18 ± 14.31	64.17 ± 9.37
*t*-value or *Z*-value	2.015	1.077	1.299	0.96	5.067	0.276	1.951	1.751	0.034	0.113	0.204
*p* value	0.047	0.284	0.196	0.339	< 0.001	0.782	0.054	0.082	0.973	0.910	0.373
Female											
Stenosis (34 cases)	2.333 ± 2.174	4.436 ± 1.643	1.537 ± 0.866	8.153 ± 4.36	7.129 ± 1.9	328.853 ± 96.610	234.735 ± 254.734	1.501 ± 0.307	132.74 ± 23.81	77.82 ± 14.29	66.45 ± 9.82
Control (58 cases)	3.671 ± 6.998	4.499 ± 0.848	1.797 ± 1.114	6.374 ± 1.593	5.647 ± 1.241	307.093 ± 86.475	154.172 ± 227.798	3.177 ± 12.006	136.22 ± 17.99	79.71 ± 14.10	64.66 ± 8.91
*t*-value or *Z*-value	1.349	0.207	1.247	2.289	4.071	1.115	1.567	0.812	0.795	0.615	0.892
*p* value	0.181	0.837	0.216	0.028	< 0.001	0.268	0.121	0.419	0.429	0.540	0.375

### Comparison of radiomics feature between the CAD group and the non-CAD group

The radiomics feature were compared between the CAD group and the non-CAD group. The analysis revealed statistically significant differences (*p* < 0.05) in Area, BSA index, Mean, Perim, IntDen, Median, RawIntDen, and Round groups. Further, a separate comparison of radiomics feature in males also showed statistically significant differences (*p* < 0.05) in Area, BSA index, Mean, Perim, IntDen, Median, RawIntDen, and Round between the CAD group and the non-CAD group. Similarly, in females, there were statistically significant differences (*p* < 0.05) in Area, BSA index, Mean, Perim, IntDen, Median, RawIntDen, and Round between the CAD group and the non-CAD group. For detailed results, please refer to Table 3.

**Table 3:** Comparison of tradiomics feature between the stenosis group and the control group

	Mean	StdDev	Mode	IntDen	Median	Skew	Kurt	RawIntDen	AR	Solidity
	First-order									
Total population										
Stenosis (97 cases)	95.641 ± 5.612	35.291 ± 5.547	80.15 ± 20.681	209,322.66 ± 116,026.911	89.64 ± 7.531	– 1.502 ± 0.325	3.379 ± 1.548	414,663.38 ± 403,780.483	1.259 ± 0.145	0.227 ± 0.114
Control (118 cases)	91.8073 ± 6.031	34.783 ± 7.631	79.98 ± 20.849	162,359.14 ± 76,732.947	82.13 ± 7.506	– 1.583 ± 0.435	3.806 ± 1.721	278,852.14 ± 124,104.909	1.246 ± 0.182	0.205 ± 0.115
*t*-value or *Z*-value	4.785	0.564	0.06	3.419	7.292	1.557	1.891	3.191	0.595	1.417
*p* value	< 0.001	0.573	0.952	0.001	< 0.001	0.121	0.06	0.002	0.552	0.158
Male										
Stenosis (63 cases)	96.544 ± 5.839	35.131 ± 5.323	82.03 ± 21.517	214,740.833 ± 111,437.712	90.89 ± 8.076	– 1.46 ± 0.326	3.266 ± 1.476	427,997.13 ± 472,339.38	1.286 ± 0.134	0.239 ± 0.127
Control (60 cases)	92.377 ± 6.413	34.573 ± 8.277	85.23 ± 20.886	178,670.351 ± 78,969.925	83.68 ± 7.514	– 1.534 ± 0.446	3.824 ± 1.755	301,298.9 ± 129,097.005	1.232 ± 0.19	0.226 ± 0.143
*t*-value or *Z*-value	3.772	0.447	0.837	2.062	5.116	1.049	1.913	2.007	1.842	0.516
*p* value	< 0.001	0.656	0.404	0.041	< 0.001	0.297	0.058	0.047	0.068	0.607
Female										
Stenosis (34 cases)	93.969 ± 4.81	35.587 ± 6.009	76.68 ± 18.848	199,283.111 ± 125,185.978	87.32 ± 5.824	– 1.58 ± 0.312	3.589 ± 1.676	389,956.74 ± 232,723.005	1.21 ± 0.153	0.205 ± 0.081
Control (58 cases)	91.219 ± 5.604	35.001 ± 6.965	74.55 ± 19.537	145,485.465 ± 71,140.303	80.52 ± 7.212	– 1.634 ± 0.421	3.786 ± 1.701	255,631.34 ± 115,259.877	1.261 ± 0.172	0.183 ± 0.072
*t*-value or *Z*-value	2.487	0.409	0.51	2.298	4.678	0.641	0.54	3.147	1.417	1.396
*p* value	0.015	0.683	0.611	0.026	< 0.001	0.523	0.591	0.003	0.16	0.166

	Area (cm^2^)	BSA	BSA index	Perim	Circ	Area%	Round
	Shape-order						
Total population							
Stenosis (97 cases)	22.661 ± 10.958	1.803 ± 0.174	12.532 ± 5.698	362.393 ± 47.195	0.208 ± 0.106	23.144 ± 11.562	0.804 ± 0.09
Control (118 cases)	14.589 ± 5.98	1.758 ± 0.184	8.244 ± 3.152	319.716 ± 50.98	0.18484 ± 0.11	20.312 ± 11.975	0.69569 ± 0.11
*t*-value or *Z*-value	6.503	1.816	6.625	6.315	1.574	1.753	7.842
*p* value	< 0.001	0.071	< 0.001	< 0.001	0.117	0.081	< 0.001
Male							
Stenosis (63 cases)	22.929 ± 10.164	1.882 ± 0.143	12.162 ± 5.066	361.728 ± 44.377	0.218 ± 0.119	24.363 ± 12.917	0.786 ± 0.082
Control (60 cases)	15.774 ± 5.893	1.886 ± 0.139	8.331 ± 3.008	325.854 ± 51.264	0.206 ± 0.136	22.523 ± 14.745	0.707 ± 0.117
*t*-value or *Z*-value	4.804	0.15	5.128	4.155	0.52	0.267	4.309
*p* value	< 0.001	0.881	< 0.001	< 0.001	0.604	0.462	< 0.001
Female							
Stenosis (34 cases)	22.166 ± 12.444	1.654 ± 0.122	13.217 ± 6.744	363.624 ± 52.699	0.189 ± 0.075	20.887 ± 8.205	0.838 ± 0.095
Control (58 cases)	13.363 ± 5.871	1.625 ± 0.119	8.154 ± 3.319	313.367 ± 50.337	0.163 ± 0.068	18.025 ± 7.67	0.684 ± 0.1
*t*-value or *Z*-value	3.879	1.134	4.096	4.489	1.765	1.684	7.247
*p* value	< 0.001	0.26	< 0.001	< 0.001	0.081	0.096	< 0.001

^*^All negative numbers except “Skew” take the absolute value

### Diagnostic efficacy analysis of clinical indicators and radiomics feature

ROC curves were plotted to evaluate the diagnostic efficacy of radiomics feature parameters, including gray-scale histogram (Mean, IntDen, Median, RawIntDen), area features (Area, BSA index, Perim, Round), and clinical indices (FPG, HbA1c, LpA), where statistically significant differences were observed between the two groups.

**Fig. 3:**
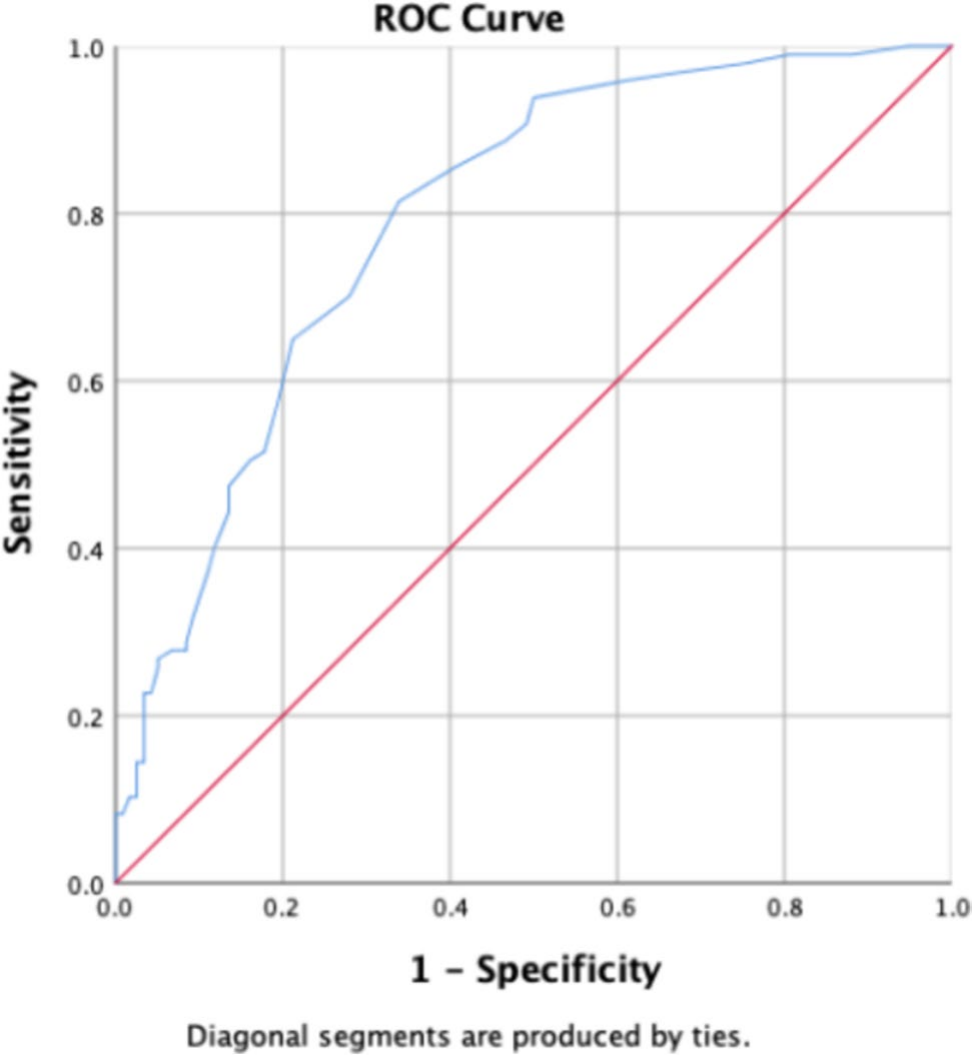
Parameter ROC curve for total population stenosis group and control group. The highest AUC values in the stenosis and control groups were obtained for the parameter “Round”

**Fig. 4:**
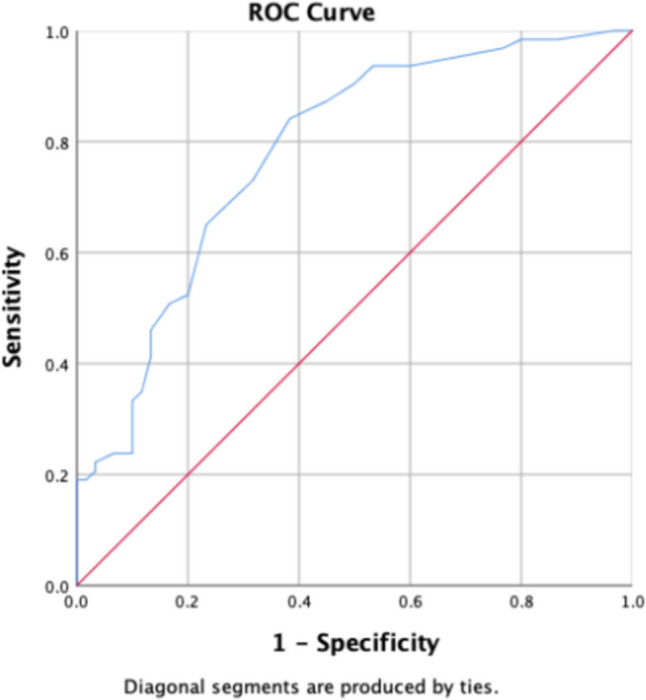
Statistically significant parametric ROC curve for the male group (BAS index). In the male group, the highest AUC values were obtained for the parameter “BSA index”

**Fig. 5:**
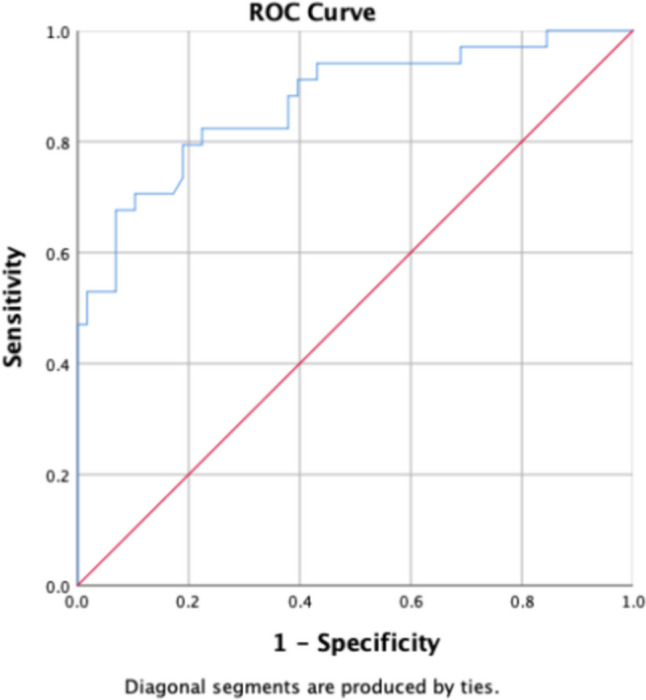
Statistically significant parameter ROC curve for the female group (Round). In the female group, the highest AUC values were obtained for the parameter “Round”

In the total population, Round showed the best diagnostic efficacy for the radiomics feature (AUC = 0.775). The male group was the Median (AUC = 0.735). Round for women group (AUC = 0.871), For detailed results, please refer to Table 4 and Figs. 3, 4, and 5.

**Table 4:** Selected radiomics feature and clinical indicators

Group	Parameter group	Feature/parameter	AUC	*p* value	95% *CI*		(level of) sensitivity	Specificity	Threshold value
					Lower	Upper			
Total population	Shape-order	Area	0.748	< 0.001	0.683	0.813	0.729	0.661	16.4879
		BSA index	0.753	< 0.001	0.687	0.818	0.5	0.881	11.69178
		Round*	0.775	< 0.001	0.714	0.836	0.719	0.72	0.7525
		Perim	0.743	< 0.001	0.677	0.808	0.74	0.653	334.754
	First-order	Median	0.753	< 0.001	0.689	0.817	0.802	0.593	83.5
		Mean	0.681	< 0.001	0.61	0.752	0.583	0.712	95.065
		IntDen	0.623	0.002	0.547	0.699	0.365	0.873	239,306.29
		RawIntDen	0.66	< 0.001	0.587	0.734	0.573	0.729	327,473
	Clinical indicators	FPG	0.571	0.074	0.494	0.648	0.885	0.237	5.08
		HbA1c	0.797	< 0.001	0.738	0.856	0.823	0.661	5.85
		LpA	0.603	0.009	0.527	0.68	0.719	0.542	63
Male	Shape-order	Area	0.734	< 0.001	0.645	0.822	0.794	0.6	16.48791
		BSA index	0.743	< 0.001	0.656	0.829	0.492	0.9	11.707458
		Round	0.697	< 0.001	0.605	0.789	0.651	0.667	0.751
		Perim	0.718	< 0.001	0.626	0.809	0.587	0.817	357.5345
	First-order	Median*	0.735	< 0.001	0.648	0.822	0.683	0.667	87.5
		Mean	0.701	< 0.001	0.608	0.793	0.587	0.75	95.87
		IntDen	0.602	0.05	0.502	0.703	0.413	0.85	239,678.685
		RawIntDen	0.635	0.01	0.537	0.734	0.603	0.7	325,082
	Clinical indicators	CRP	0.596	0.067	0.495	0.696	0.556	0.633	1.835
		HbA1c	0.781	< 0.001	0.7	0.863	0.841	0.617	5.85
Female	Shape-order	Area	0.764	< 0.001	0.666	0.861	0.912	0.5	11.15587
		BSA index	0.773	< 0.001	0.676	0.871	0.882	0.534	7.387823
		Round*	0.871	< 0.001	0.793	0.949	0.676	0.931	0.8145
		Perim	0.767	< 0.001	0.669	0.864	0.765	0.724	335.477
	First-order	Median	0.773	< 0.001	0.675	0.871	0.882	0.586	80.5
		Mean	0.637	0.029	0.521	0.752	0.706	0.534	92.01
		IntDen	0.63	0.038	0.508	0.752	0.5	0.759	191,744.4
		RawIntDen	0.684	0.003	0.569	0.799	0.529	0.793	365,048
	Clinical indicators	HbA1c	0.81	< 0.001	0.723	0.896	0.941	0.534	5.45

## Discussion

In this study, we investigated the efficacy of EAT radiomics feature and some clinical indicators for assessing coronary atherosclerotic stenosis at the level of the transverse left coronary bifurcation on chest CT. We also differentiated between male and female subgroups to examine parameter differences. The results showed statistically significant differences in several parameters, covering three aspects: EAT gray-scale histogram (Mean, IntDen, Median, RawIntDen), EAT area (Area, BSA index, Perim, Round), and clinical indices (HbA1c, ApoA, FPG).

**Fig. 6:**
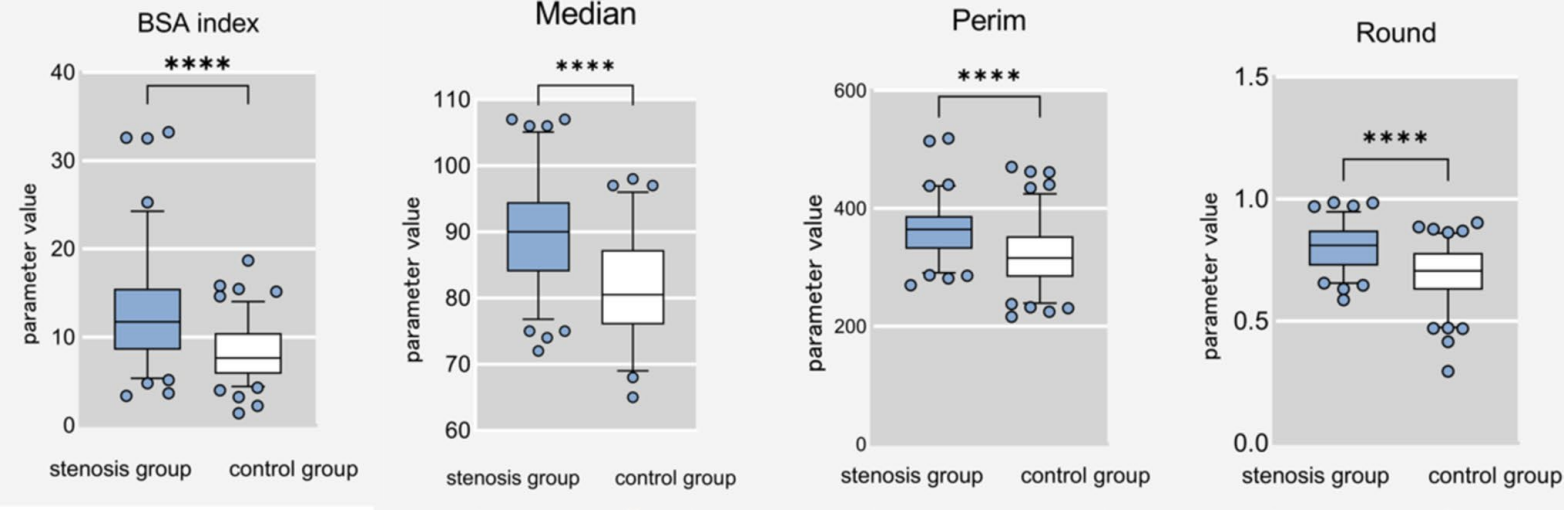
Graphs showing the results of BSA index, Median, Perim, Round. Comparison of four parameters “BSA index, Median, Perim, Round” in the stenosis group and the control group

The gray-level histogram belongs to the first-order features of radiomics feature, also known as intensity features [16]. It is related to the frequency distribution of gray levels within the ROI and depends on a single voxel value. The four with statistically significant differences are: Mean (the average gray value), Median (the median gray value), IntDen (the product of area and average gray value), and RawIntDen (the sum of all pixel values in the ROI). Due to the threshold setting of these four parameters, measurements are negative, and the absolute value of the comparisons of these is taken to be greater for the narrower group (*p* < 0.05).From the perspective of the first-order feature in Fig. 2, without taking the absolute value, both the CAD group and the non-CAD group were located on the left side of the origin, and the grayscale value of the CAD group was farther and more to the left of the origin, showing a more obvious difference from that of the non-CAD group. Based on the results, if the absolute value of Round is greater than 0.7525, the high risk of CAD should be considered, and the greater the value, the more likely. This finding can alert radiologists or clinicians to the possibility of coronary artery stenosis in patients. From a biological perspective, this can be explained as follows: at the cellular level, the hypertrophy of adipocytes leads to hypoxia caused by the reduction of capillary density, which is prone to EAT necrosis and triggers an inflammatory response due to its gapless contact with the coronary arteries, thus inducing coronary atherosclerosis [17]. Increased EAT density in patients with obstructive coronary artery disease (50% coronary artery stenosis) may lead to increased fat density [18], but this fibrosis is not sufficient to affect the decreased density from adipocyte proliferation and hypertrophy. Additional study [19] showed that a significant difference in EAT-attenuation index between patients with and without coronary artery disease, with the fat attenuation index correlating with coronary stenosis > 50%. West et al. [20] performed deep learning on coronary computed tomography angiography (CCTA) images and found that EAT volume was associated with coronary artery disease, suggesting that EAT should be used as the gold standard for detecting unhealthy visceral fat. There was a good correlation between EAT left-main coronary artery cross-sectional area and EAT volume [14], justifying our selection of this level for measurement to ensure reproducibility. This study demonstrated that radiomics-based analysis can quantify CT images of EAT, with Round being most associated with coronary stenosis. The formula for Round is 4 × area/(π × ROI long axis^2^), and the diagnostic efficacy was the highest in both the total population and female population, suggesting that Round may be an important parameter for the assessment of coronary artery stenosis (Fig. 6).

Considering the effect of body size on EAT area, we compared the BSA index and found that the BSA index had stronger diagnostic efficacy than EAT area in both the total population and male and female populations, potentially providing new insights for subsequent studies.

This study reveals an association between blood glucose levels and coronary stenosis. Type 2 diabetics exhibit higher levels of EAT compared to non-diabetics [21]. In patients with severe coronary artery atherosclerosis, systemic glucose/insulin metabolism disorders and decreased serum lipocalin were identified as significant independent factors contributing to the intensity of oxidative stress in EAT adipocytes [22]. Furthermore, EAT demonstrated a positive correlation with the insulin resistance index, with higher EAT thickness observed in insulin-resistant cases compared to controls [23].The findings of the present study are consistent with these previous reports, suggesting that elevated blood glucose and insulin resistance induce oxidative stress, leading to reduced antioxidant capacity, endothelial damage, impaired blood supply within the EAT fat matrix, and exacerbation of hypoxia and ischemia. Consequently, these factors trigger inflammatory reactions and contribute to the development of coronary atherosclerosis. However, it is important to note that blood glucose levels can be significantly influenced by medications and other factors, which may warrant further investigation and subgroup analysis in future studies.

Gender is considered one of the traditional risk factors for coronary heart disease, with men having a higher risk compared to women of the same age [24]. This higher risk in men is attributed to the predominance of visceral fat, which contributes to inflammatory lesions, affects metabolism, and leads to obesity [25]. Cardiac fat, in particular, exhibits a pronounced effect [26]. The results of this study show that the diagnostic efficacy of the female population was higher than that of the male population across all three parameters: gray-scale histogram, shape-order feature, and clinical indicators.

EAT is strongly associated with cardiovascular dysfunction and cardiovascular disease, making noninvasive imaging-based techniques to observe the quality, distribution, and function of EAT critical for cardiovascular risk reduction, and potentially integrated into clinical practice in the future [27].Expanding the reach of clinical data without burdening patients, radiomics feature utilizing chest-CT images holds high clinical value for identifying coronary artery disease.

## Limitations

The study has following limitations:

(1) small sample size; (2) studies from a single center; (3)analysis was limited to only one level of the left coronary artery trunk bifurcation, and full-domain first-order or more advanced features was not conducted, warranting further investigation; (4)Non-ECG gated CT, was unable to completely avoid motion artifacts.

## Conclusion

Despite of these limitations, the findings of this study suggest that chest CT, as a routine examination, is feasible for conducting radiomics feature on its images to assess EAT. Multiple can be employed to evaluate the risk of coronary stenosis, providing valuable information for predicting coronary artery disease. Furthermore, this assessment holds greater significance in prompting clinical diagnosis and treatment, particularly among females compared to males.
